# CRISPR-Cas9-Mediated Knockout of *MLO3* Confers Enhanced Resistance to Reniform Nematode in Upland Cotton

**DOI:** 10.3390/plants14223491

**Published:** 2025-11-15

**Authors:** Foster Kangben, Sonika Kumar, Anqi Xing, Li Wen, Wei Li, Stephen Parris, John Lawson, Zhigang Li, Lauren Carneal, Meredith Cobb, Robert L. Nichols, Christina Wells, Paula Agudelo, Churamani Khanal, Christopher A. Saski

**Affiliations:** 1 Department of Plant and Environmental Sciences, Clemson University, Clemson, SC 29634, USA; fkangbe@g.clemson.edu (F.K.); sonikak@clemson.edu (S.K.); superwenli@163.com (L.W.); liwei90314@163.com (W.L.); sparri2@clemson.edu (S.P.); johndlawson320@gmail.com (J.L.); zhiganl@clemson.edu (Z.L.); lecarneal@gmail.com (L.C.); mac112699@gmail.com (M.C.); pagudel@clemson.edu (P.A.); ckhanal@clemson.edu (C.K.); 2 A&L Scientific Editing Inc., Durham, NC 27709, USA; anqi.xing1984@gmail.com (A.X.); cewells85@gmail.com (C.W.); 3 Cotton Incorporated, Cary, NC 27513, USA

**Keywords:** cotton, *Mildew Resistance Locus O* (*MLO*), Clustered Regularly Interspaced Short Palindromic Repeats (CRISPR), host-plant resistance, *Rotylenchulus reniformis*

## Abstract

Upland cotton (*Gossypium hirsutum* L.) is a major global commodity crop whose production is threatened by the reniform nematode (*Rotylenchulus reniformis* Linford and Oliveira), a plant-parasitic pest that causes substantial yield losses. Host-plant resistance offers a sustainable management strategy, but currently available resistant cotton cultivars provide only partial protection and often require supplemental control methods. In this study, Clustered Regularly Interspaced Palindromic Repeats (CRISPR)–CRISPR-associated 9 (Cas9) gene editing was used to generate targeted knockouts of *Mildew Resistance Locus O* (*GhiMLO3*) in cotton and assess its role in resistance to *R. reniformis*. Four independent knockout lines (A1, D3, E1, and P3) were developed, confirmed by sequencing, and evaluated for nematode resistance under controlled greenhouse conditions. Nematode reproduction was significantly reduced on lines D3 and E1, with lower egg counts and fewer vermiform life stages compared with the control genotypes, Coker 312 (WT), Delta Pearl, and Jin668. The edited lines also showed characteristic mesophyll cell-death phenotypes, suggesting potential pleiotropic effects associated with *MLO*-mediated resistance. Sequence analysis confirmed multiple homozygous and heterozygous mutations in *MLO3* alleles from both the A and D subgenomes, with D3 and E1 lines displaying the strongest resistance profiles. These findings demonstrate that *MLO3* gene editing is a promising approach for improving *R. reniformis* resistance in cotton.

## 1. Introduction

Upland cotton (*Gossypium hirsutum* L.) is the world’s leading natural-fiber crop and an economically important commodity. The United States produces about one-third of the world’s cotton exports, primarily in the southeastern “Cotton Belt,” which offers ideal growing conditions [1]. However, cotton production is threatened by plant-parasitic nematodes. The reniform nematode (*Rotylenchulus reniformis* Linford and Oliveira) is a major pathogen with a severe economic impact on cotton production [2,3,4]. *R. reniformis* infestations result in substantial economic losses, with documented annual losses exceeding USD 130 million in the United States [5]. Field studies have demonstrated that this parasitic nematode can reduce cotton yield up to 64% in infected fields, making it a significant threat to domestic cotton production [6,7]. *R*. *reniformis* accounts for approximately 11% of pest-related agricultural losses, affecting over 50 economically significant crops across various climates [7,8]. *R*. *reniformis* was first reported on cotton and cowpea by Smith and Taylor [9]. They observed a high level of infestation caused by an unidentified pathogen [9], which was later named the “reniform nematode” in reference to the kidney-shaped bodies of the adult females [10]. *R. reniformis* is an obligate parasite with infective vermiform females and nonparasitic males [11]. Management of *R. reniformis* is challenging because of its short life cycle (17 days from egg to egg at 27–32 °C), rapid reproductive capacity (multiple generations, 5–7 per year), ability to colonize deeper soil layers beyond the reach of nematicides, and capacity for anhydrobiosis [7]. During anhydrobiosis, desiccated *R. reniformis* stages remain viable for prolonged periods without a host [4,12,13]. These adaptive traits have facilitated the dominance of *R. reniformis* in the southeastern U.S. Cotton Belt. Addressing the production constraints imposed by *R. reniformis* requires multiple strategies, including the identification of resistance genes and targeted breeding to develop resistant genotypes for sustainable cotton production [14,15,16,17].

Cotton varieties with moderate resistance to reniform nematode have been developed [18]. Although these varieties offer protection and can reduce nematode populations, they may not completely prevent infestation or damage, particularly under intense nematode pressure [19]. Consequently, additional management practices such as crop rotation and nematicide application are often necessary for effective nematode management. Integrating the use of resistant varieties with other management practices is necessary to extend the durability of resistance [20,21].

Current management primarily involves host-plant resistance and chemical treatments [22]. Host-plant resistance derived from wild cotton species like *Gossypium longicalyx*, *Gossypium barbadense*, and *Gossypium aridum* is an effective, sustainable, and economically viable strategy that leverages inherent plant defenses while preserving the soil microbial environment [11,23,24,25]. In particular, the wild cotton *G. longicalyx* confers strong resistance via hypersensitive responses [24,26,27]. However, its diploid nature, creeping growth habit, and adaptability to drier conditions, as well as the need for crossbreeding and identification of homozygous lines with the required resistance, necessitate years of meticulous evaluation [19,24,28].

In recent years, significant progress has been made in developing upland cotton cultivars that exhibit partial resistance or tolerance to *R. reniformis* [29]. However, repeated cultivation of resistant cultivars may encourage the emergence of virulence and the evolution of nematode populations capable of overcoming these resistance mechanisms [30,31]. To mitigate this risk, it is essential to gain a deeper understanding of host-plant resistance mechanisms, including specific genes that act as negative regulators of plant immunity. Understanding these resistance mechanisms will support breeding efforts aimed at the development of highly resistant cotton cultivars for sustainable nematode management.

*Mildew Resistance Locus O* (*MLO*) encodes a kinase-like, calmodulin-binding protein expressed in various plant tissues, including reproductive organs, that participates in plant defense and cell death regulation. MLO proteins play crucial roles in developmental and immune responses, with expression patterns often linked to reproductive development and pathogen defense [32,33,34]. Silencing or knockout of specific *MLO* genes provides broad-spectrum resistance to powdery mildew across multiple plant species, as first reported in barley [35]. In *Arabidopsis*, simultaneous knockout of *AtMLO2, AtMLO6*, and *AtMLO12,* which are co-orthologs of barley *MLO*, confers resistance to the powdery mildew pathogens *Golovinomyces orontii* and *Golovinomyces cichoracearum* [36,37,38,39,40,41]. These three co-orthologs exhibit partial functional redundancy, with *AtMLO2* triggering broad-spectrum antifungal resistance [41]. *MLO* genes have been reported in cotton, but little is known about their functions in immunity, including their potential roles in powdery mildew susceptibility, phytohormone signaling, and defense against other pathogens, including plant-parasitic nematodes [42]. Although cotton cultivars with partial *R. reniformis* resistance are available, continued breeding and development are essential to counteract evolving nematode resistance. Recent advances in genome-editing technologies, particularly Clustered Regularly Interspaced Palindromic Repeats (CRISPR)–CRISPR-associated 9 (Cas9), have enabled targeted mutagenesis in cotton via *Agrobacterium tumefaciens*-mediated hypocotyl transformation, resulting in the production of stable transgenic plants with desired agronomic and resistance traits [30,43,44,45]. These mutations may be homozygous, affecting both alleles of the target gene, or heterozygous, affecting only one allele, and each mutation type has distinct implications for trait expression and inheritance [46].

In a preliminary study on cotton *MLO* genes, *GhiMLO3* (118566) and *GhiMLO1* (136200 & 14552) were identified as functional homologs of *AtMLO2*, *AtMLO6*, and *AtMLO12* in *Arabidopsis*. Using CRISPR–Cas9 gene editing, we generated four sequence-validated *mlo3* knockout lines in the Coker 312 background. The objectives of this study were to assess the resistance of the *mlo3* knockout lines to *R. reniformis* and to confirm successful gene editing at the *GhiMLO3* locus.

## 2. Results

### 2.1. Identification and Sequence Validation of MLO3 Knockout Lines in the F_2_ and F_3_ Generations

Sequencing of five F_2_ individuals each from the A1, D3, E1, and P3 lines showed high CRISPR–Cas9 editing efficiency of *MLO3* in the A and D subgenomes (Table 1), with multiple mutations identified. Across the four F_2_ families, two mutation types were detected at the first gRNA target site (homozygous insertions and deletions) and three mutation types at the second gRNA target site (homozygous insertions and deletions and heterozygous deletions) (Table 1). Only mutations consistently detected across replicates were considered genuine and are reported in Table 1 and Table 2.

In the A1 knockout population, mutations in *MLO3* (A) and *MLO3* (D) were observed in three F_2_ plants. At the first gRNA target site in *MLO3* (A), two plants exhibited a homozygous “A” insertion. At the second target site, one plant had a heterozygous “CT” deletion, and another showed a homozygous “CT” deletion. For *MLO3* (D), three F_2_ plants displayed a homozygous “GG” deletion at the first target site, and two plants exhibited a homozygous “T” insertion at this site. At the second target site, three plants had a 15 bp homozygous deletion, and two had a homozygous “TT” insertion (Table 1).

All five D3 F_2_ plants contained a 16 bp insertion at the first target site in *MLO3* (A); four plants also contained a 60 bp deletion at the second target site, and one plant contained a 25 bp deletion at the second target site. Four of the five D3 F_2_ plants carried a 75 bp deletion at the first target site and a 147 bp deletion at the second target site in *MLO3* (D). All mutations identified in the D3 family resulted in premature stop codons in the predicted MLO proteins (Table 1).

In the E1 population, one F_2_ plant had an “A” insertion at the first target site and a “CT” deletion at the second target site in *MLO3* (A). For *MLO3* (D), the same individual had a “T” insertion at the first target site and a “TT” insertion at the second target site. The remaining four E1 F_2_ plants had the same 16 bp homozygous mutation in *MLO3* (A) but showed two different *MLO3* (D) mutation profiles (Table 1). All E1 mutations resulted in premature stop codons and large deletions in the predicted MLO3 proteins.

In the P3 population, mutations were identified in both subgenomes. In *MLO3* (A), three plants carried a homozygous “A” insertion (Table 1) at the first target site and a homozygous “CT” deletion at the second target site. The remaining two plants had a large homozygous 404 bp deletion in *MLO3* (A). For *MLO3* (D), four plants exhibited a large homozygous deletion of 507 bp, and one plant lacked a detectable band. All mutations identified in *MLO3* (D) were homozygous, whereas both homozygous and heterozygous mutations were found in *MLO3* (A), suggesting different editing mechanisms of Cas9 in the A and D subgenomes.

**Figure 1:**
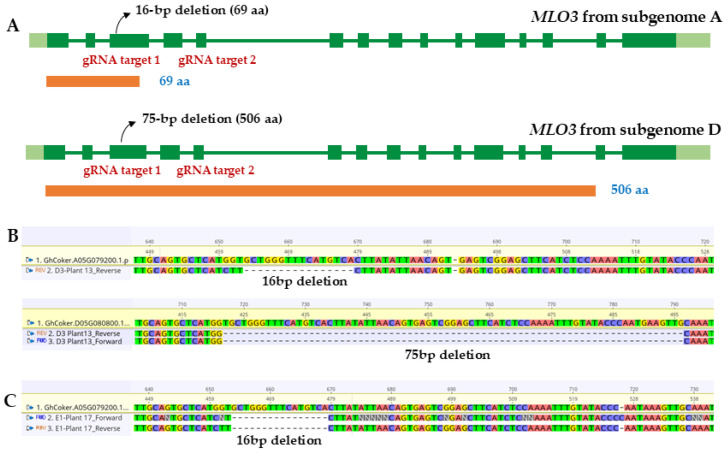
Characterization of CRISPR/Cas9-mediated modifications in *MLO3* homeologs from the D3 and E1 cotton lines: (**A**) Schematics of *MLO3* A and D subgenomes, respectively, showing CRISPR/Cas9-mediated modifications present in event D3 plant 13 and E1 plant 17 from the F_3_ generation. (**B**) Sequence alignment of *MLO3* A subgenome (top) and *MLO3* D subgenome (bottom) from WT Coker and D3 plant 13, showing 16 bp and 75 bp deletions, respectively. (**C**) Sequence alignment of *MLO3* A subgenome from WT Coker and E1 plant 17, showing a 16 bp deletion.

On the basis of the F_2_ genotyping results, we advanced the D3 and E1 *mlo3* knockout lines to the F_3_ generation, as these lines had homozygous mutations (*MLO3* A and D subgenomes) in 4–5 of the plants genotyped. Twenty-four seeds each for E1 and D3 were advanced to the F_3_ generation, and genotyping of the F_3_ plants revealed various mutations, including insertions, deletions, and substitutions. Figure 1A shows details of the CRISPR/Cas9-mediated modifications in *MLO3* (A) and *MLO3* (D) from two plants in the F_3_ generation: D3 plant 13 and E1 plant 17.

D3 plant 13 had a 16 bp deletion in the third exon of *MLO3* A subgenome at target site 1 that led to a premature stop codon and a predicted truncated protein of 69 amino acids. It also carried a 75 bp deletion in the third exon of *MLO3* D subgenome, which resulted in a truncated protein of 506 amino acids (Figure 1B). E1 plant 17 also had an identical 16 bp deletion in the third exon of *MLO3* A subgenome that led to a truncated protein of 69 amino acids (Figure 1C). However, its *MLO3* D subgenome sequence exhibited partial replacement by downstream sequences, indicative of larger rearrangements (Table 2). Some mutants had complex replacements or substitutions within the targeted region. Genotyping also identified allelic differences between homeologous loci, with specific mutations present only in the A or D subgenome copies of *GhiMLO3*. Overall, these mutations confirmed the successful CRISPR-Cas9 editing of the *GhiMLO3* locus.

### 2.2. Phenotyping

**Figure 2:**
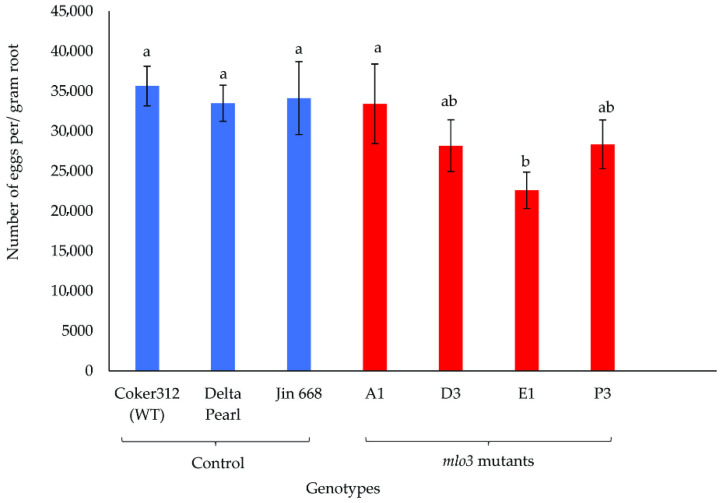
Egg production of *R. reniformis* on roots of seven cotton genotypes inoculated with 1000 freshly hatched juveniles and harvested 60 days after inoculation. Data from two experiments are combined and are presented as mean ± SE of ten replicates. Treatment means that share lowercase letters are not significantly different (Student’s *t*-test, *p* ≤ 0.05).

**Figure 3:**
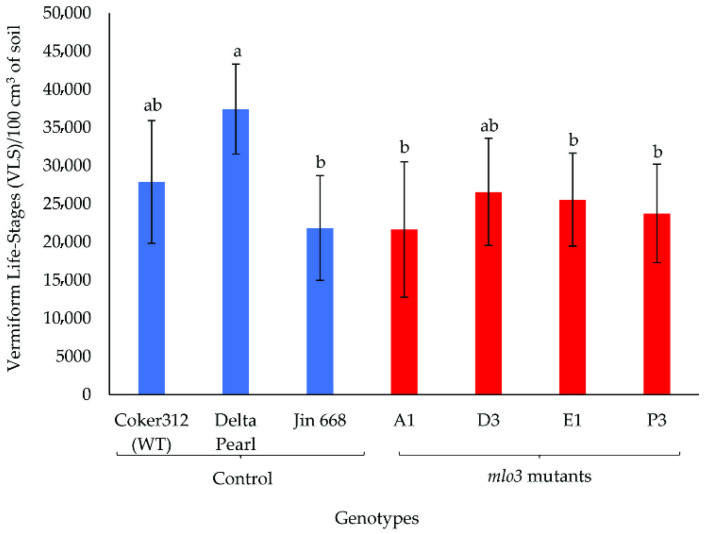
Reproduction of *R. reniformis* assessed as the number of vermiform life stages per 100 cm^3^ of soil. Seven cotton genotypes were inoculated with 1000 freshly hatched juveniles and harvested 60 days after inoculation. Data from two experiments are combined and are presented as the mean ± SE of ten replicates. Treatment means that share lowercase letters are not significantly different (Tukey’s HSD, *p* ≤ 0.05).

**Figure 4:**
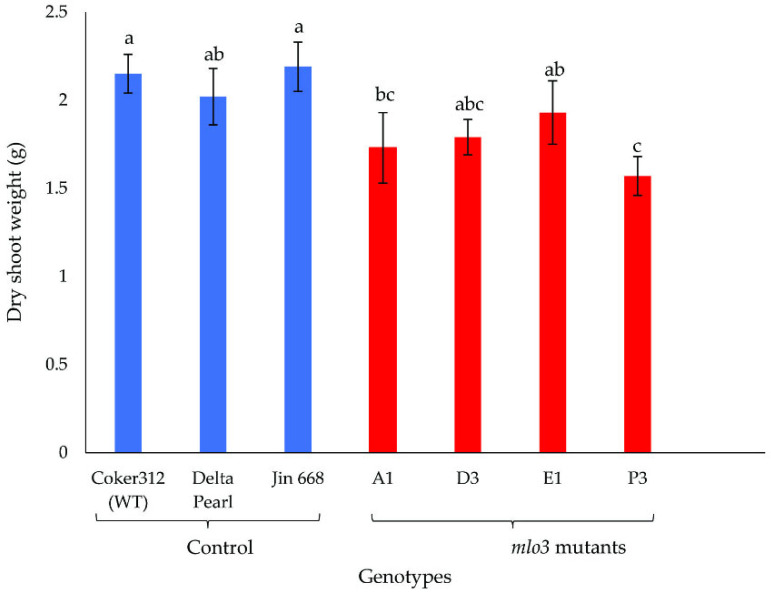
Shoot dry weights of seven cotton genotypes 60 days after inoculation with 1000 freshly hatched *R. reniformis* juveniles. Data from two experiments were combined and are presented as the mean ± SE of ten replicates. Treatment means that share lowercase letters are not significantly different (Student’s *t*-test, *p* ≤ 0.05).

Two greenhouse experiments were performed to evaluate the resistance of the transgenic cotton lines (A1, D3, E1, and P3) and three non-transgenic control lines to the reniform nematode. Data from the two experiments were pooled for analysis, as there were no significant interactions between genotype and experiment. Genotype had a significant effect on nematode reproduction, as evidenced by significant differences among genotypes in egg number (*p* = 0.0354) and number of vermiform life stages (VLS, *p* = 0.0155). There was also a significant effect of genotype on shoot biomass (*p* = 0.0027) but not on root biomass (*p* = 0.3294) (Figure 2, Figure 3 and Figure 4).

Nematode reproduction ranged from 22,558 to 35,646 eggs per gram of root and was consistently lower on *mlo3* mutants than on the control genotypes Coker 312 (WT), Delta Pearl, and Jin668 (Figure 2). In particular, egg numbers were significantly lower on E1 *mlo3* mutant plants than on any of the control cultivars.

The number of VLSs per 100 cm^3^ of soil ranged from 21,637 to 37,406 (Figure 3) and was significantly lower in three of the *mlo3* knockout lines than in the Delta Pearl controls: A1 (21,637), E1 (25,555), and P3 (23,724). The Coker 312 and Jin668 controls had fewer VSLs and did not differ significantly from the *mlo* knockouts (Figure 3).

**Figure 5:**
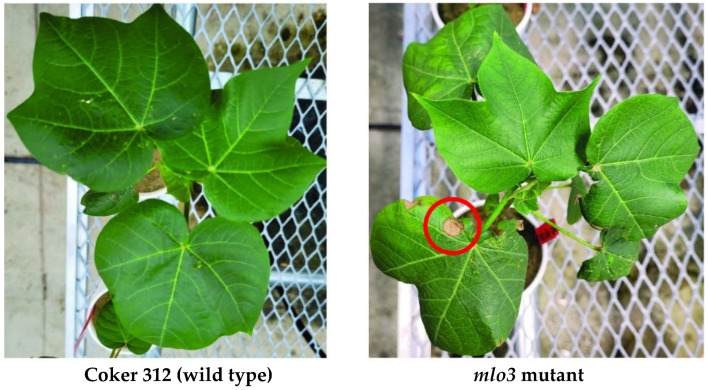
Representative macroscopic infection phenotypes at 4 weeks after infection of Coker 312 (wild type) and the D3 *mlo3* mutant. The red circle highlights a necrotic leaf lesion.

Cotton shoot dry weight ranged from 1.57 g to 2.15 g and was significantly influenced by genotype (Figure 4). In general, shoot dry weight was higher in Coker 312, Delta Pearl, and Jin 668 than in the *mlo3* knockouts, likely owing to segregation among the knockout genotypes (Figure 4). However, the shoot biomass of the D3 and E1 knockout lines did not differ significantly from that of the control genotypes. Interestingly, the *mlo3* knockout lines occasionally displayed a characteristic leaf lesion phenotype (Figure 5), suggestive of the mesophyll cell death commonly associated with *MLO* gene mutations.

The reproductive dynamics of a nematode species within a host crop serve as a reliable indicator of the host’s susceptibility to that nematode. The results from the greenhouse study revealed significant genotype-dependent variations in nematode reproduction (Figure 2, Figure 3 and Figure 4). Specifically, the knockout lines E1, D3, and P3 displayed ~15–30% reductions in egg production relative to control lines (Figure 2), and the A1, E1, and P3 lines showed significant reductions in VLS numbers relative to Delta Pearl (Figure 3). These reductions in nematode reproduction likely reflect loss-of-function mutations that impaired the function of MLO3 in plant defense signaling. Across many plant species, MLO proteins are known to suppress basal (natural) immunity, enabling pathogen invasion. Therefore, knockout or disruption of MLO3 may have reduced this immune suppression, enhancing activation of defense responses such as upregulation of stress-responsive genes, strengthening of cell-wall integrity, and/or increased production of antimicrobial compounds. Our initial transcriptomic profiling provided further support for this hypothesis, as *MLO3*, among other *MLO* homologs in *G. hirsutum* (Coker 313), consistently exhibited a susceptibility-associated expression profile, indicating that it may contribute to modulating host–pathogen interactions and therefore to *R*. *reniformis* vulnerability in cotton.

## 3. Discussion

This study demonstrates that CRISPR–Cas9 knockout of *GhiMLO3* in upland cotton can increase its resistance to *R. reniformis*. MLO proteins have been studied extensively for their roles in resistance to fungal pathogens in various crops, and recent research has highlighted the strong potential of *mlo*-mediated resistance against powdery mildew [38,47,48]. Nonetheless, recent studies suggest a coevolutionary arms race between host plants and pathogens, with evidence that the powdery mildew pathogen *Blumeria hordei* can overcome the broad-spectrum resistance conferred by barley *mlo* loss-of-function mutations [49]. However, the potential involvement of *MLO* genes in resistance to plant-parasitic nematodes remains largely unexplored. To investigate the involvement of *MLO* genes in *R*. *reniformis* infection, we performed an initial evaluation of cotton *MLO* gene expression in response to reniform nematode inoculation. Transcriptomic profiling of cotton roots infected with *R*. *reniformis* revealed differences in the expression patterns of *MLO* homologs. *MLO3*, in particular, exhibited consistently higher expression from 3 to 12 days after inoculation (DAI). This temporal expression pattern closely reflected the histological development of nematode infection, from early penetration through the cortical and endodermal layers to the establishment of mature syncytia. The concurrence of *MLO3* induction and syncytial development suggests a potential role for *MLO3* in facilitating the host cellular reprogramming required for the formation and maintenance of the nematode feeding site. By contrast, *MLO1*-like homologs were transiently induced during the initial stages of infection (3–9 DAI), but their expression declined as the syncytia matured. This transient activity may reflect a role in early signaling or defense modulation, whereas the persistent expression of *MLO3* implies a more sustained role in the structural or physiological remodeling of host cells. These findings prompted us to perform targeted knockout of *MLO3* to assess its involvement in reniform nematode susceptibility. Our findings provide new insights into the potential function of MLO proteins in defense against nematodes, expanding our understanding of host-plant resistance mechanisms in cotton.

Sequencing of the *mlo3* knockout mutants revealed both homozygous and heterozygous mutations, including insertions and deletions (indels) at the first and second guide RNA target sites (Table 1). Specifically, sequencing analysis identified various mutations, including single-nucleotide polymorphisms and indels, across the A and D subgenomes in the five analyzed F_2_ knockout lines. For example, the D3 line contained a 16 bp deletion in *MLO3* (A) and a 75 bp deletion in *MLO3* (D), whereas E1 carried only the 16 bp deletion in *MLO3* (A) (Table 1).

Genotyping of D3 plant 13 and E1 plant 17 from the F_3_ generation revealed various mutations at the *GhiMLO3* locus, including small insertions, deletions, complex rearrangements, and truncated non-functional proteins. Allelic variation was observed between the A- and D-subgenome copies in D3 plant 13 and E1 plant 17 (Figure 1 and Table 2), including partial replacement by downstream sequences. Overall, sequencing confirmed successful CRISPR–Cas9 editing and the generation of knockout lines suitable for downstream phenotypic characterization.

Targeted mutagenesis has been used to manipulate *MLO* homologs in various plant species, conferring enhanced resistance to powdery mildew. CRISPR–Cas9 editing of wheat *MLO* genes produced loss-of-function alleles by inducing specific deletions that resulted in truncated, non-functional MLO proteins, consistent with our results in cotton. Such engineered deletions led to robust powdery mildew resistance [50,51,52]. Likewise, disruption of *AtMlo2* significantly improved resistance in *Arabidopsis thaliana*, but loss-of-function mutations in *AtMLO6* or *AtMLO12* alone did not yield comparable results. Mutations in all three genes (*AtMLO2*, *AtMLO6*, and *AtMLO12*) conferred complete resistance to powdery mildew. Such targeted gene edits can accelerate functional genomic studies, enabling detailed investigation into the roles of the edited genes in plant growth, development, disease resistance, and stress responses. Despite the ongoing segregation in the F_2_ generation of *mlo3* transgenic lines, some plants exhibited resistance or tolerance, displaying significantly reduced numbers of eggs and nematodes. The D3 and E1 transgenic lines of the F_2_ generation emerged as promising candidates for *MLO*-mediated nematode resistance, as both lines showed consistent phenotypic and genotypic results and did not differ from control lines in shoot dry weight.

The *mlo3* knockout lines generally showed lower *R. reniformis* reproduction than the control genotypes, indicating enhanced resistance to nematode infection. These results align with previous reports on the involvement of *MLO* genes in plant defense responses, highlighting the potential application of *MLO*s for the development of host-plant resistance strategies [53]. The known role of MLO proteins in regulating plant defense and cell death pathways may explain the observed reduction in nematode populations in the *mlo3* knockout lines [48,53,54]. Moreover, the observed variability in nematode resistance among the different *mlo3* knockout lines suggests that specific *mlo3* alleles may differentially affect resistance levels, highlighting the necessity for further research [55]. Developing such resistant cultivars could offer a sustainable approach for managing reniform nematode infestations, potentially reducing reliance on nematicides, promoting agricultural productivity, and maintaining soil health [50].

Two of the *mlo3* knockout lines (F_2_) exhibited lower shoot biomass than the controls, which may partly reflect segregation-driven variation among the *mlo3* lines. This reduction is consistent with prior reports that *MLO*-mediated resistance can impose a growth–defense trade-off whereby resources are reallocated from biomass accumulation toward defense responses [37,56,57]. Moreover, the *mlo3* knockout lines also displayed necrotic leaf lesions, a phenotype commonly associated with *MLO* mutations. This suggests that the activation of cell death pathways may further influence plant development, consistent with previous reports [47,48,49]. However, confirming the involvement of programmed cell death will require histological or microscopic validation in future studies.

Previous studies have documented pleiotropic effects associated with *MLO* mutations, including low grain yield, chlorosis, necrosis, premature leaf senescence, and reduced growth rates, often linked to the spontaneous formation of callose-containing cell appositions [58,59]. These effects could also be attributed to off-target effects of CRISPR gene editing, which might account for the sterility of the T_0_ Coker 312-derived mutant plants generated by transformation, suggesting that *MLO* disruption may have broader physiological consequences. However, these drawbacks are potentially compensated for by a reduction in nematode reproduction. Consistent with previous reports [58,59,60,61], we also observed leaf chlorosis and necrosis in some transgenic lines, specifically noting chlorotic and necrotic spots predominantly on the adaxial surfaces of leaves (Figure 5).

Our findings confirm that loss-of-function of the *MLO3* locus results in suppression of *R. reniformis* populations, highlighting the role of *MLO3* in host susceptibility. Evaluations of resistant cultivars typically involve comparisons with susceptible cultivars under conditions in which nematicides are also applied, reflecting real-world agricultural management practices [29]. Although nematicides were not used in this study, the *mlo3* transgenic lines showed substantial reductions in reniform nematode reproduction compared with controls, indicating their strong potential for use in nematode management without chemical intervention. These results underscore the potential of *MLO3* and related alleles in breeding programs aimed at the development of nematode-resistant crops. Nevertheless, the observed trade-offs between resistance and plant growth highlight the necessity for further research to identify *MLO3* alleles that provide robust resistance without reducing productivity.

To further assess the effectiveness of these CRISPR-induced mutations, a comparative study involving the F_4_ generation of these lines and commercial cultivars, both resistant and susceptible, should be performed. In parallel, detailed analyses of root architecture and histology during nematode infection would provide deeper insights into the mechanisms underlying resistance. Future investigations should also include field-based evaluations of homozygous *mlo3* knockout lines (D3 and E1) to examine their resistance to reniform nematodes and other economically significant plant-parasitic nematodes under production conditions.

## 4. Materials and Methods

### 4.1. Generation and Characterization of Mlo3 Knockouts

Using the Golden Gate Reaction cloning system and *AarI* digestion methods, two gRNAs (Table S1) targeting cotton *GhiMLO3*, together with the *Arabidopsis thaliana* U6 promoter (AthU6 promoter) and the guide RNA scaffold from the pMOD_B2515 35S:SpCas9-nos cassette from pMOD_A0101, were subcloned into pTRANS_210 to generate the final construct. Primers were designed, and ~1 kb of DNA downstream of the ATG start codon of *MLO3* was sequenced. The results showed that the *MLO3* sequences of Coker 312 were identical to those of TM-1 in the genome database. The sgRNA Scorer 2.0 web tool (https://frederick.cancer.gov/resources/repositories/sgrnascorer (accessed on 1 April 2018) was used to identify target sites with different activities. The two predicted *GhiMLO3* gRNAs had off-target scores of 44 and 66 and CrisprScan scores of 56 and 60, which fell within the acceptable range for genome-editing applications. Two target sites were chosen as the editing position in *MLO3* and edited in a single transformation.

We used the commonly referenced and well-annotated Coker 312 genotype because of its known regenerative ability [46,62]. Owing to pollen sterility, the *mlo3* T_0_ plants were crossed with wild-type (WT) Coker 312 to generate F_1_ seeds. The resulting F_1_ progeny were self-pollinated to produce F_2_ seeds. F_2_ plants derived from these crosses (lines A1, D3, E1, and P3) were genotyped to identify CRISPR–Cas9-induced mutations within the guide-RNA target regions in *MLO3* from both subgenomes. To confirm the presence of these mutations, both alleles (A and D subgenomes) were amplified, cloned, and sequenced for each edited line. For each *mlo3* knockout line, mutations were confirmed in at least three cloned amplicons. Leaf tissues were collected from each line for the extraction of genomic DNA, and DNA samples were amplified by PCR using specific primers (Table S1). PCR products were cloned and sequenced following standard Sanger sequencing protocols at the Genomics Core, Biosciences at Arizona State University. Sequencing chromatograms were analyzed using Geneious Prime software (version 2023.2.1). The characterized transgenic lines were then screened for resistance against reniform nematodes. On the basis of genotyping results from the F_2_ generation, two *GhiMLO3* knockout lines (E1 and D3) were advanced to the F_3_ generation and genotyped to identify plants carrying homozygous mutations.

### 4.2. Reniform Nematode Resistance Assessment of Mlo3 Knockout Lines

Two experiments were performed in summer and fall in a greenhouse to evaluate the resistance of the transgenic cotton lines (A1, D3, E1, and P3) to reniform nematode. Three controls, Coker 312 (wild type), Jin668 (a cotton line amenable to regeneration), and Delta Pearl (a commercial cultivar), were included. Wagram loamy sand soil was collected from a soybean field at the Edisto Research and Education Center in South Carolina and steam-sterilized at 275 °F (135 °C) for three hours. Before use, the soil was tested to ensure the absence of any plant-parasitic nematodes.

### 4.3. Establishment of Experiments

Plants were grown in styrofoam cups (473.2 mL) containing 0.625 kg (625 cm^3^) of soil. Treatments included seven cotton genotypes: four *mlo3* lines and three controls. Five seeds of a single genotype were planted in each pot, and after 13 days, the pots were thinned to one healthy seedling per genotype. Plants were grown in the greenhouse (cooling/heating set points were 25/32 °C), with a 16 h light/8 h dark photoperiod (>300 μmol m^−2^ s^−1^ light) and watered (100 mL) manually every day, with the amount of water adjusted on the basis of soil moisture. Each pot contained a single healthy plant, and pots were arranged in a randomized complete block design with five replicates per treatment/genotype (35 total pots per experiment). The pots were rotated on the benches at weekly intervals to minimize positional effects. The experimental design was consistently applied across both experiments, resulting in a combined sample size of 10 plants per genotype. Standard fertilization and insect management practices were followed [63]. Fourteen days after sowing, each plant was inoculated with 1000 freshly extracted juvenile reniform nematodes suspended in 1 mL of tap water. For inoculation, three 2 cm depressions were created in a triangular pattern around each seedling and injected with inoculum using a sterilized 1-mL pipette tip. The experiments were terminated 60 days post inoculation. Each plant shoot was cut above the soil level, and dry biomass was recorded after oven drying the shoots at 42 °C for seven days. Soil and roots were carefully removed from the pots, and the roots were washed to eliminate soil particles. Eggs were extracted from the roots by agitating whole root systems in 0.6% NaOCl for four minutes [64]. Nematodes were extracted from a 100-cm^3^ subsample of soil from each pot using the centrifugal-flotation technique [65].

Eggs were counted within 24 h of extraction using a compound microscope (American Optical Company, Southbridge, MA, USA) at 40× magnification. Soil samples were stored in a walk-in cold room at 4 °C until nematode extraction (no more than one week). Extraction and enumeration were performed within two weeks of experiment termination.

### 4.4. Data Analysis

Data were analyzed using one-way analysis of variance (ANOVA) with the mixed model procedure in JMP PRO 16.0 (SAS Institute, Cary, NC, USA). Before analysis, nematode reproduction data were log-transformed to satisfy the normality assumption of ANOVA. Genotype was treated as a fixed effect and replication as a random effect. Post hoc mean comparisons were performed using Student’s *t*-test (*p* ≤ 0.05) and Tukey’s HSD (*p* ≤ 0.05).

## Figures and Tables

**Table 1:** Mutations identified in the *MLO3* genes from subgenomes A and D in four F_2_ transgenic lines.

Transgenic Individual	Mutations in *MLO3* (A)		Mutations in *MLO3* (D)	
	gRNA1	gRNA2	gRNA1	gRNA2
A1: 1	A insertion (HM)	CT deletion (HM)	T insertion (HM)	TT insertion (HM)
A1: 2–4	No PCR band		GG deletion (HM)	TGCTCACTATGGCTT deletion (HM)
A1: 5	A insertion (HM)	CT deletion (HT)	T insertion (HM)	TT insertion (HM)
D3: 1–3	A insertion; 16-bp insertion	60-bp deletion (HT)	75-bp deletion (HM)	147-bp deletion (HM)
D3: 4	A insertion; 16-bp insertion	60-bp deletion (HT)	No PCR band	
D3: 5	A insertion; 16-bp insertion	25-bp deletion (HT)	75-bp deletion (HM)	147-bp deletion (HM)
E1: 1	A insertion (HM)	CT deletion (HM)	T insertion (HM)	TT insertion (HM)
E1: 2	16-bp deletion (HM)	60-bp deletion (HM)	No PCR band	
E1: 3–5	16-bp deletion (HM)	60-bp deletion (HM)	T insertion (HM)	TT insertion (HM)
P3: 1	404-bp deletion (HM)		507-bp deletion (HM)	
P3: 2 & 3	A insertion (HM)	CT deletion (HM)	507-bp deletion (HM)	
P3: 4	A insertion (HM)	CT deletion (HM)	No PCR band	
P3: 5	404-bp deletion (HM)		507-bp deletion (HM)	

HM, homozygous; HT, heterozygous. A1: 1–5 (line A1, plants 1–5).

**Table 2:** Mutations identified in the *MLO3* genes from subgenomes A and D in two F_3_ transgenic lines.

Transgenic Individual	Mutations in *MLO3* (A)		Mutations in *MLO3* (D)	
	Guide RNA1	Guide RNA2	Guide RNA1	Guide RNA2
D3: 13	16-bp deletion	replaced and substituted	75-bp deletion (HM)	replaced and substituted
E1: 17	16-bp deletion and A insertion	A insertion	extra A insertion	extra AA insertion

## Data Availability

Data are contained within the article or Supplementary Material.

## Supplementary Materials

The following supporting information can be downloaded at: https://www.mdpi.com/article/10.3390/plants14223491/s1, Table S1: Primers used in this research for PCR.

## Abbreviations

The following abbreviations are used in this manuscript

*MLO*	Mildew Resistance Locus O
*GhiMLO3*	Mildew Resistance Locus O from *G. hirsutum*
CRISPR-Cas9	Clustered Regularly Interspaced Short Palindromic Repeats (CRIPSR)–CRISPR-associated protein 9
gRNAs	Guide RNAs
*AtMLO*	*Arabidopsis thaliana Mildew Resistance Locus O*
WT	Wild type
VLS	Vermiform life-stage
